# Nonoperative treatment for nonperforated appendicitis in children: a systematic review and meta-analysis

**DOI:** 10.1007/s00383-019-04610-1

**Published:** 2019-12-14

**Authors:** Sonia Maita, Björn Andersson, Jan F. Svensson, Tomas Wester

**Affiliations:** 1 Pediatric Surgery Unit, Department of Women’s and Children’s Health, University of Padua, Padua, Italy; 2 Department of Pediatric Surgery, Karolinska University Hospital, 171 76 Stockholm, Sweden; 3 Department of Women’s and Children’s Health, Karolinska Institutet, Stockholm, Sweden

**Keywords:** Nonoperative treatment, Antibiotics, Appendectomy, Nonperforated, Appendicitis, Children

## Abstract

Acute appendicitis is the most common surgical emergency in children. Nonoperative treatment of nonperforated acute appendicitis in children is an alternative to appendectomy. The purpose of this systematic review and meta-analysis was to determine the outcomes of nonoperative treatment of nonperforated acute appendicitis in children in the literature. Databases were searched to identify abstracts, using predefined search terms. The abstracts were reviewed by two independent reviewers and articles were selected according to inclusion and exclusion criteria. Data were extracted by the two reviewers and analyzed. The literature search yielded 2743 abstracts. Twenty-one articles were selected for analysis. The study design was heterogenous, with only one randomized controlled study. The symptoms resolved in 92% [95% CI (88; 96)] of the nonoperatively treated patients. Meta-analysis showed that an additional 16% (95% CI 10; 22) of patients underwent appendectomy after discharge from initial hospital stay. Complications and length of hospital stay was not different among patients treated with antibiotics compared with those who underwent appendectomy. Nonoperative treatment of nonperforated acute appendicitis children is safe and efficient. There is a lack of large randomized controlled trials to compare outcomes of nonoperative treatment with appendectomy.

## Introduction

### Rationale

Appendicitis is the most common indication for emergency surgery in both adults and children worldwide. The estimated lifetime risk is 7–8% and the peak incidence is in the second decade of life [1, 2].

Operative management has been the gold standard of treatment for many years based on the assumption that, left untreated, acute appendicitis would always progress to perforation. This assumption has been questioned and today, there is strong support for spontaneous resolution of appendicitis [2]. Nonoperative treatment of inflammatory and infectious diseases in the abdomen has been proposed and favored for other conditions like diverticulitis [3]. Nonoperative treatment for acute nonperforated appendicitis has been proven to work well in the short term [4]. No long-term data have been presented.

Despite both open and laparoscopic appendectomy being regarded as low-risk and effective procedures, operative management is associated with risks and complications. The risks are those associated with general anesthesia and surgical complications such as hemorrhage, surgical site infection, injury to surrounding structures, ileus, adhesive small bowel obstruction, the potential need for reoperation or negative appendectomy. Advantages of the initial nonoperative treatment strategy are the avoidance of complications related to surgery and anesthesia, but these risks should be balanced against the risk of complications related to antibiotic treatment and recurrent appendicitis. Apart from balancing the risk of surgery against nonoperative treatment, there may be other reasons for not to undergo surgery. The appendix is a reservoir for bacteria that normally constitute the gut flora, and is needed to recolonize the bowel after bacterial infections, e.g. diarrheal disease [5]. A biofilm, adherent colonies of microbes growing within an extracellular matrix, is most prominent in the appendix and decreases progressively to the distal end of the bowel [5]. The vermiform appendix is capable of producing mesenchymal stem cells [6]. Hypothetically, the vermiform appendix is a reservoir for stem cells capable of bowel repair throughout life.

Furthermore, the use of both imaging [7] and biomarkers [8, 9] makes it easier to accurately differentiate perforated from nonperforated acute appendicitis and to guide the decision-making.

### Objective

The aim of this study was to systematically review the available evidence of nonoperative treatment of acute nonperforated appendicitis in the pediatric population and to compare the efficacy and safety of nonoperative versus surgical treatment.

## Methods

### Protocol and registration

This systematic review and meta-analysis were conducted according to the Preferred Reporting Items for Systematic Reviews and Meta-analyses (PRISMA) statement.

### Eligibility criteria

All studies focusing on the initial nonoperative management and comparing antibiotic treatment with appendectomy for acute nonperforated appendicitis in children were eligible for inclusion.

Commentaries, correspondence, editorials, letters, clinical guidelines, surveys, and case reports, as well as studies reporting nonoriginal data (systematic reviews, meta-analyses, narrative reviews) were excluded.

Studies addressing both adults and children or both perforated and nonperforated appendicitis and that did not report data from nonperforated disease and children separately were excluded. We also excluded all studies reporting data about immune-compromised children because of the potentially worse outcomes.

### Information sources

A systematic search of the literature was performed in Medline via PubMed, Embase, Cochrane and Web of Science in May 2019.

### Search strategy

Using Boolean operators AND and OR, we used all possible combination of the following search terms: "acute appendicitis, appendicitis, uncomplicated, noncomplicated, nonperforated, unperforated, conservative, antibiotic, nonoperative, child, children, infant, pediatric, adult”. The search was limited to human subjects and children (0–18 years), but without language or publication year restriction.

### Study selection

The online systematic review management program Covidence (https://www.covidence.org/) was used to coordinate the screening and data collection process. Preliminary screening of all studies on title and abstracts was performed independently by two reviewers (SM and BA) with any disagreement resolved by the senior reviewer (JFS). After initial screening, full-text articles, meeting inclusion criteria, were selected for inclusion.

### Data collection process

The data extraction was performed independently by two investigators (SM and BA) after reading the full-text publications. Both researchers extracted data into predefined protocols. The datasets were compared and any irregularities were corrected by a joint assessment among the authors. The final protocol specified 38 data items.

### Data items

The main outcomes were the efficacy of nonoperative treatment and the complication rate of both treatment strategies. Efficacy was defined as no need of appendectomy during the initial hospital stay. A third outcome was appendectomy, in children who were discharged from the initial hospital stay, due to recurrent appendicitis or recurrent abdominal pain with normal appendix. Interval appendectomies due to surgeon’s or parents’ choice were also included.

We defined complications as conditions requiring general anesthesia after the initial treatment; perforated appendicitis or peritonitis after nonoperative treatment or postoperative complications requiring invasive intervention. Negative appendectomy was included as a complication in patients undergoing appendectomy as initial treatment.

Additional outcomes were hospital stay during the initial admission and total hospital stay, which included readmission during follow-up.

### Summary measures

The summary effects of each respective meta-analysis were presented in forest plots, rendering standardized mean differences (SMD) or odds ratio (OR) with 95% confidence intervals (CI), respectively.

## Results

### Study selection

**Fig. 1:**
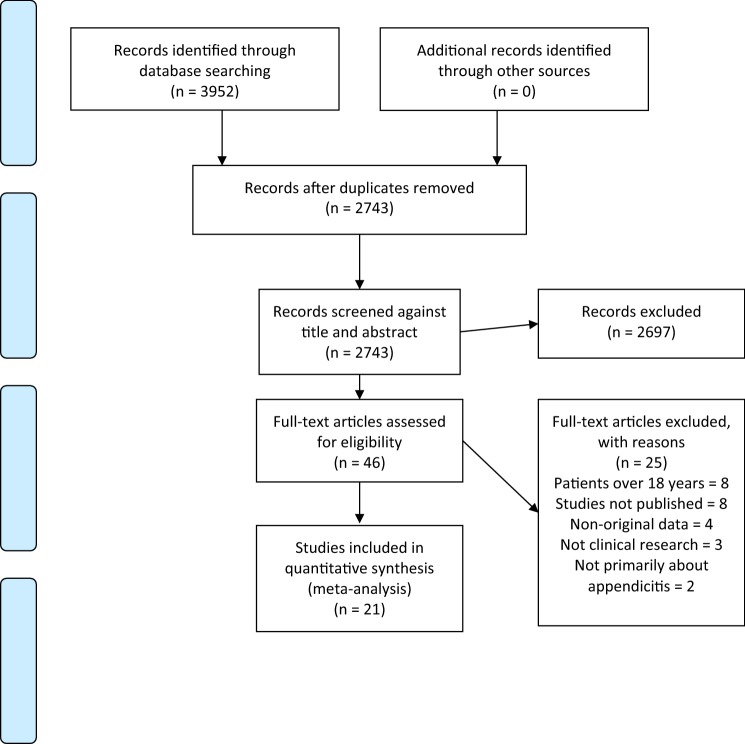
Prisma flowchart showing the process of selecting articles for analysis

The process to select articles is shown in the PRISMA flowchart in Fig. 1. The literature search yielded 2743 abstracts, which were screened. Forty-six articles were reviewed and 21 of these were included in the final analysis. The number of articles included in the separate quantitative analyses depended on availability of data for each outcome measure in each of the studies.

### Study characteristics

The studies are briefly summarized in Table 1 [10-30]. Seven of the included studies were retrospective and 14 were prospective. Thirteen of the studies included comparative data, while 8 studies reported outcomes of nonoperative management without a control group. One of the comparative studies was based on administrative data from 45 hospitals in the United States [22]. Several of the comparative studies described selection based on patient or parent choice [17–20, 25]. There was only one randomized controlled trial, which was a pilot trial. The pilot trial was designed to generate data to inform future randomized controlled trials [16]. In total, the studies reported 5727 patients treated nonoperatively. The studies had different inclusion and exclusion criteria with respect to age, symptom duration, and the presence of an appendicolith.

**Table 1:** Characteristics of included studies

Study	Year of publication	Study design	Patients *n*, NOT	Patients *n*, appendectomy	Follow-up
Kaneko et al. [1]	2004	Prospective, noncomparative	22	–	Median 36 months (24–45)
Abes et al. [2]	2007	Retrospective, noncomparative	16	–	12 months
Armstrong et al. [3]	2014	Retrospective, comparative	12	12	Median 6.5 months
Koike et al. [4]	2014	Retrospective, comparative	130	114	Mean 30.6 months
Gorter et al. [5]	2015	Prospective, noncomparative	25	–	8 weeks
Steiner et al. [6]	2015	Prospective, noncomparative	45	–	14 months
Svensson et al. [7]	2015	Randomized controlled trial	24	26	At least 12 months
Tanaka et al. [8]	2015	Prospective, comparative	78	86	Median 4.5 years
Hartwich et al. [9]	2016	Prospective, comparative	24	50	Mean 14 months
Mahida et al. [10]	2016	Prospective, comparative	5	9	12 months
Minneci et al. [11]	2016	Prospective comparative	37	65	Median 21 months
Caruso et al. [12]	2017	Prospective, noncomparative	197	-	–
Bachur et al. [13]	2017	Retrospective, comparative^a^	4190	61522	12 months
Steiner et al. [14]	2017	Prospective, noncomparative	197	–	18 months
Mudri et al. [15]	2017	Retrospective, comparative	26	26	3 years
Lee et al. [16]	2018	Prospective, comparative	51	32	Median 13 months
Gorter et al. [17]	2018	Prospective, comparative	25	19	25 months (16-36)
Scott et al. [18]	2018	Retrospective, noncomparative	50	–	Median 305 days (125–375)
Abbo et al. [19]	2018	Retrospective, noncomparative	166	–	Median 18.8 months (13–270)
Steiner et al. [20]	2018	Prospective, noncomparative	362	–	22 months (6–43)
Knaapen et al. [21]	2019	Prospective, noncomparative	45	–	25 months (16–36)

*NOT* nonoperative treatment, *SD* standard deviation

^a^Data from an administrative database.

### Results of studies

#### Efficacy of nonoperative treatment

**Fig. 2:**
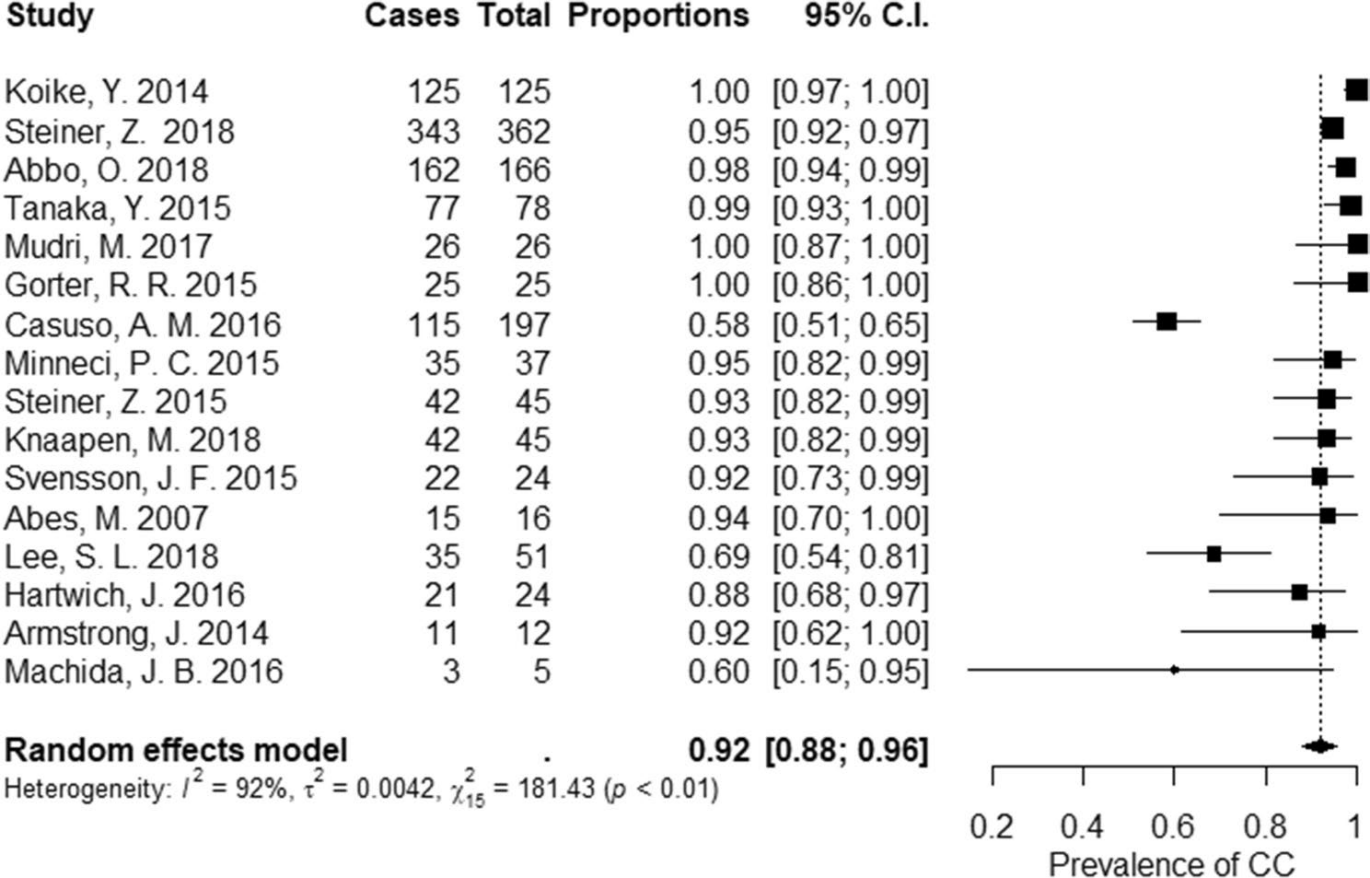
Treatment efficacy. Sixteen studies were included in the analysis of treatment efficacy, defined as discharge without further complications

Data from 16 studies were included in a meta-analysis to assess efficacy of nonoperative treatment (Fig. 2). Efficacy was defined in different ways in the included studies and nonoperative treatment was reported to be efficient in 92% [95% CI (88; 96)] of patients. Mahida et al. prospectively studied nonoperative treatment in patients with an appendicolith and reported that failure rate was 60%. The study was stopped due to patient safety concerns [19]. Others also reported an increased failure rate in patients with an appendicolith [25, 27]. Patients with an appendicolith were excluded from some studies, whereas the increased risk for failed treatment in the presence of an appendicolith could not be confirmed in other studies [16, 28].

#### Complications of nonoperative treatment

**Fig. 3:**
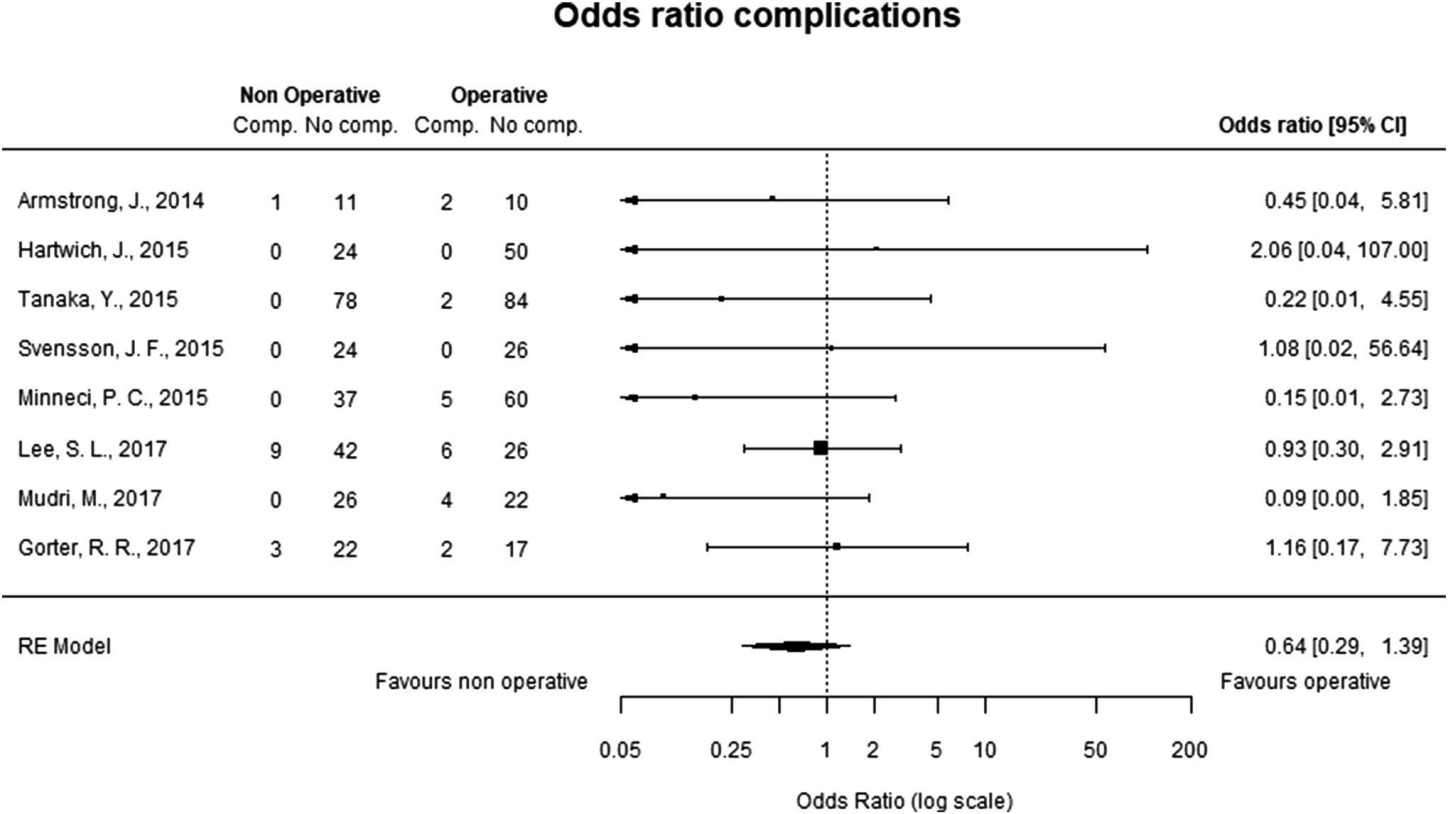
Complications. Eight studies reported complications. Negative appendectomy was included among complications in the appendectomy group

Eight studies were included in the comparative analysis of complications shown in Fig. 3. The overall complication rate was low in both groups. There were no differences with respect to complications between patients undergoing nonoperative treatment and appendectomy. Negative appendectomy, which was considered a complication in the appendectomy group, was reported in 0–6.2% of patients. A large retrospective review of administrative data from 45 pediatric hospitals in the United States showed that nonoperatively treated patients had more emergency department visits and hospitalizations compared with those managed with appendectomy [22].

#### Recurrent appendicitis and long-term outcomes

**Fig. 4:**
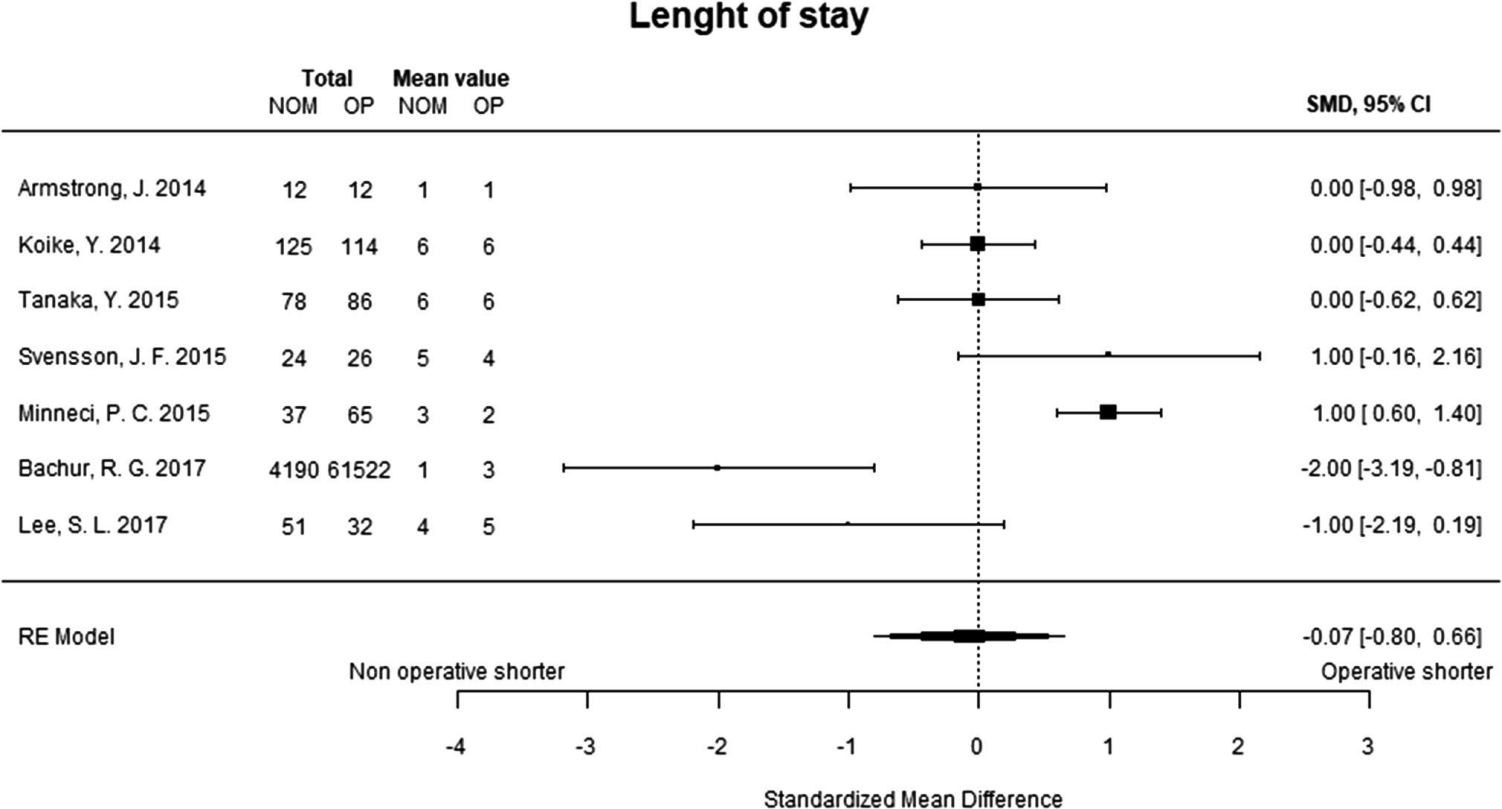
Length of hospital stay. Length of initial hospital stay was compared based on data from seven studies

Except for 8% of patients undergoing appendectomy during initial hospital stay, meta-analysis, including 21 studies, showed that 16% (95% CI 10; 22) of patients had undergone an appendectomy during follow-up (Fig. 4). The figures included patients who had undergone appendectomy due to recurrent appendicitis and patients with recurrent abdominal pain with histologically normal appendix. Svensson et al. reported that 7 of the 22 successfully nonoperatively treated patients underwent appendectomy after discharge from initial hospital stay. Only one of these patients had a microscopically confirmed recurrent acute appendicitis. The other patients underwent appendectomy due to recurrent abdominal pain (*n* = 5) or parental wish (*n *= 1) [16]. Abbo et al. reported that 2 of 19 patients had normal appendix [28]. We choose to also include patients who underwent interval appendectomy in the analysis as this required general anesthesia and could, therefore, be considered a failure.

#### Length of hospital stay

**Fig. 5:**
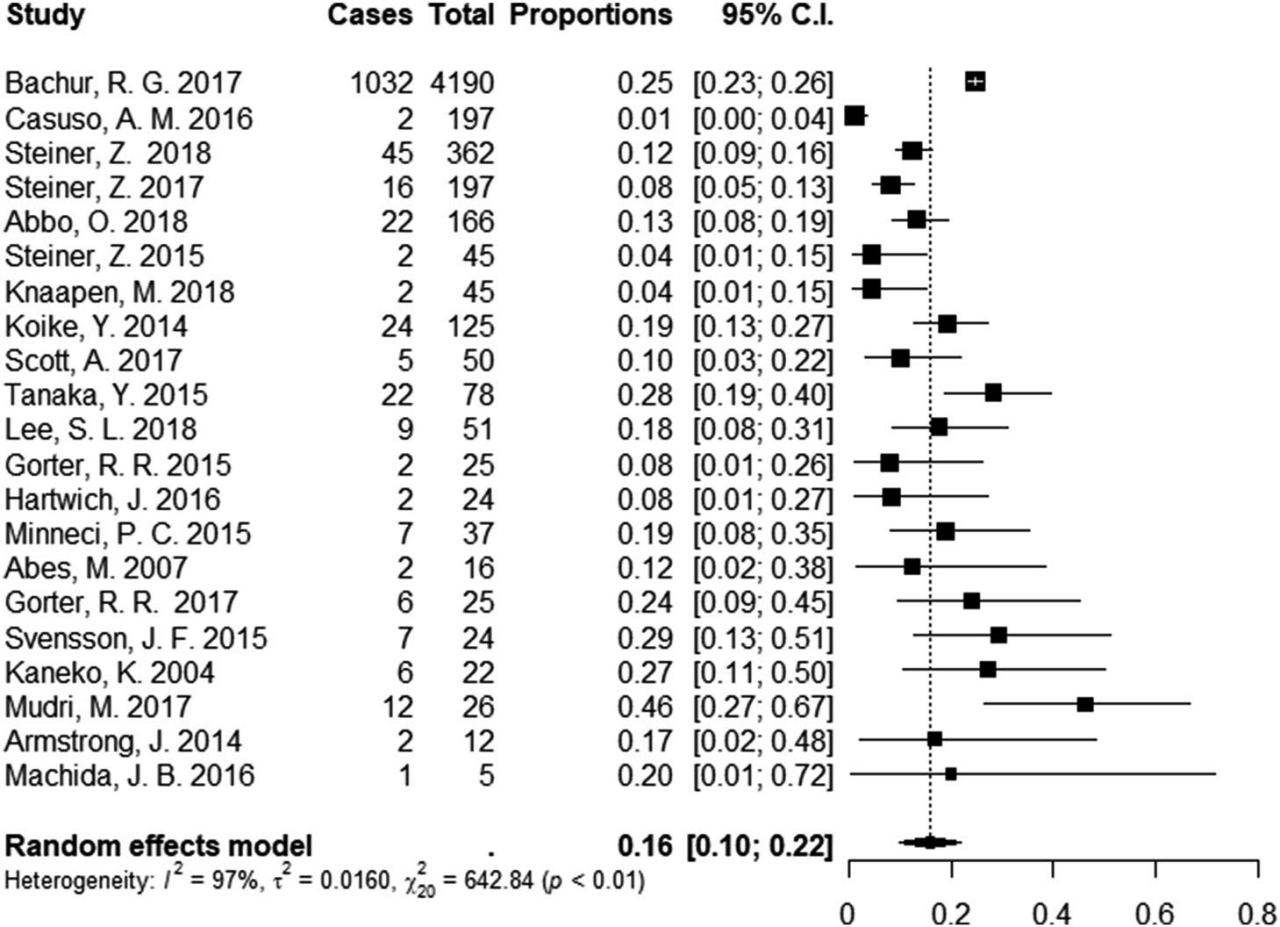
Recurrent appendicitis. Twenty-one studies were included in the meta-analysis of recurrent appendicitis after discharge from the initial hospital stay. The analysis also included patients who underwent appendectomy due to recurrent abdominal pain with normal appendix, and interval appendectomies

The length of initial hospital stay was analyzed based on data from seven studies. There was no difference between the nonoperative treatment group and appendectomy group (Fig. 5).

#### Length of total hospital stay

**Fig. 6:**
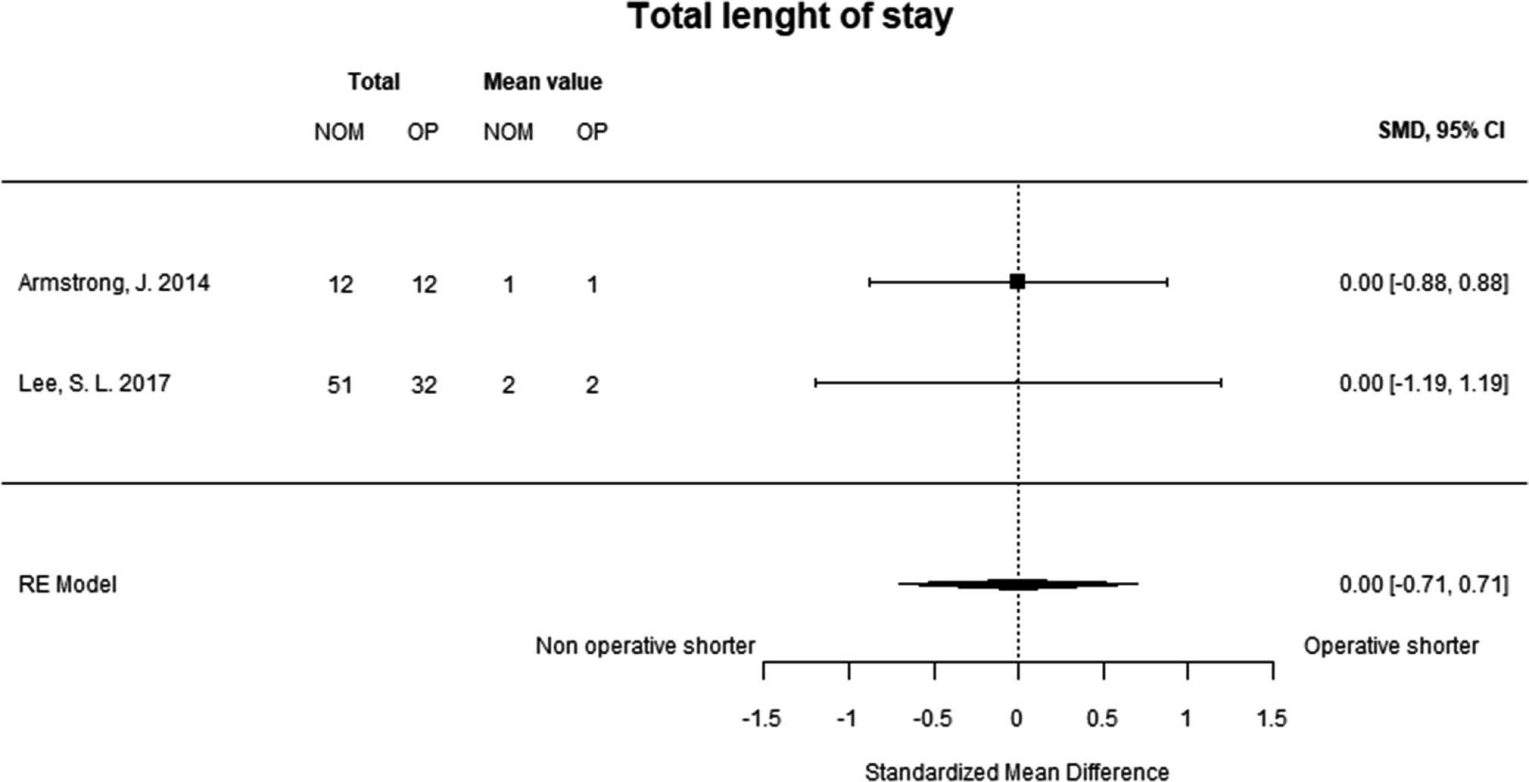
Total length of hospital stays. This included the initial hospital stay and hospital stay during readmissions

Only two studies were included in analysis of total hospital stay, which included initial hospital stay and hospital stay at readmission. There was no difference between the groups (Fig. 6).

## Discussion

### Summary of evidence

Nonoperative treatment of acute appendicitis in children is safe, with a complication rate comparable to that in patients undergoing appendectomy. The treatment is successful, defined as resolution of symptoms and discharge from initial hospital stay without appendectomy, in 92% of the patients. After discharge, another 16% of patients undergo appendectomy due to recurrent acute appendicitis or recurrent abdominal pain with normal appendix. The hospital stay is similar in patients treated nonoperatively and with appendectomy.

Kessler et al., in a previous systematic review and meta-analysis, including five comparative studies, presented data in favor of appendectomy with respect to efficacy of nonoperative treatment and readmission rate, while complications were similar in both groups. The conclusion from this study was that appendectomy should be considered the treatment of choice for management of nonperforated appendicitis in children [31]. Georgiou et al. used slightly different inclusion criteria in another recent systematic review and meta-analysis. They included comparative studies, but also studies reporting outcomes after nonoperative treatment without a control group. Nonoperative treatment was reported to be successful in 97% of the children. The hospital stay was shorter after appendectomy. Complication rate was similar in both groups. At last follow-up, 82% of patients treated nonoperatively had not undergone appendectomy, compared to 76% of patients in the present study. Fourteen percent of the patients underwent appendectomy for recurrent appendicitis [32].

Kessler et al. compared nonoperatively treated patients with and without appendicolith in a sub-analysis. No statistically significant differences were found with respect to complications and efficacy, but readmission rate was lower in those without an appendicolith. Excluding patients with appendicolith improved efficacy in the total group of nonoperatively treated patients [31].

Several studies reported on interval appendectomies done due to parent’s request [16, 18, 26] and a higher rate of emergency department visits for complaints of abdominal pain in patients who underwent nonoperative treatment [22]. One issue that may need to be further addressed is the quality of life and parent/patient satisfaction with nonoperative treatment. Four studies compared quality of life through questionnaires given to patients and their parents. Tanaka et al. showed that 1 year after initial treatment, patients who underwent operative treatment were much more satisfied than patients who underwent nonoperative treatment [17], while Hartwich et al. found experiences in favour of nonoperative treatment at 30 days [18]. Other studies found no differences between operative and nonoperative treatments [20, 25]. Minneci et al. stressed the importance of engaging families in the decision, allowing therapy to be aligned with their preferences and real-life concerns, such as cultural values and distance from the hospital [20].

## Limitations

One main limitation of this meta-analysis was the heterogenous design of included studies. Inclusion and exclusion criteria, age, symptom duration, and the presence of appendicolith, were different in the included studies. The protocol for treatment with antibiotics, in the nonoperative treatment group, varied between the studies. Several studies were of low quality, with considerable risk for selection bias. There was only one randomized controlled pilot trial available.

Several of the comparative studies used a parent or patient choice design. We appreciate that the opinion of patients and parents is crucial for nonoperative treatment for children with acute appendicitis being a viable treatment option. The balance between optimizing recruitment and achieving acceptability of a randomized controlled trial to participants is a challenge for researchers. To have high external validity, the proportion of the study population of interest who are actually recruited into the randomized controlled trial should be high and representative. But, it is also important to have high internal validity to avoid selection bias, and this is best achieved with a randomized controlled trial. Due to the lack of randomization, and the likelihood of bias toward the less severely ill patients choosing antibiotic treatment, we are not able to assume that the group undergoing appendectomy are in a similar clinical condition using the parent or patient choice design. Only a true randomized controlled trial will minimize the likelihood of these differences influencing trial results. [33] Another limitation to this meta-analysis is the limited data on long-term follow-up. In children, there are no available long-term data. In adults, a recent 5-year follow-up of the APPAC trial showed that 27% of patients had undergone appendectomy at 1 year and 39% at 5 years, indicating that recurrences occur also in long term [34].

## Future perspectives

Consequences that are related to antibiotic resistance should be taken into account when appendicitis is treated nonoperatively, but this issue has not been addressed in the present studies. Interestingly, two recent trial study protocols describe one trial planning to compare antibiotics with placebo for nonperforated appendicitis in adults and another trial designed to investigate microbiota and effects of antimicrobial treatment of appendicitis in adults. [35, 36]

## Conclusions

This systematic review and meta-analysis show that nonoperative treatment of nonperforated acute appendicitis in children is safe and efficient. However, large randomized controlled trials are necessary to compare outcomes with appendectomy. Currently, the authors only treat nonperforated appendicitis with antibiotics as part of an ongoing randomized controlled trial. Patients not included in the trial undergo appendectomy.
